# Motives for choosing, switching and stopping daily or event‐driven pre‐exposure prophylaxis – a qualitative analysis

**DOI:** 10.1002/jia2.25389

**Published:** 2019-10-14

**Authors:** Hanne ML Zimmermann, Sanne W Eekman, Roel CA Achterbergh, Maarten F Schim van der Loeff, Maria Prins, Henry JC de Vries, Elske Hoornenborg, Udi Davidovich, On behalf of the Amsterdam PrEP Project team in the HIV Transmission Elimination AMsterdam Consortium (H‐TEAM)

**Affiliations:** ^1^ Department of Infectious Diseases, Research and Prevention Public Health Service of Amsterdam Amsterdam the Netherlands; ^2^ Department of Infectious Diseases Public Health Service of Amsterdam STI Outpatient Clinic Amsterdam the Netherlands; ^3^ Department of Infectious Diseases Amsterdam Institute for Infection and Immunity (AI&II) Amsterdam UMC University of Amsterdam Amsterdam the Netherlands; ^4^ Department of Dermatology Amsterdam Institute for Infection and Immunity (AI&II) Amsterdam UMC University of Amsterdam Amsterdam the Netherlands

**Keywords:** counseling, HIV, prevention, men who have sex with men, PrEP, sexual behaviour

## Abstract

**Introduction:**

In settings where both daily and event‐driven pre‐exposure prophylaxis (PrEP) are offered to men who have sex with men (MSM), a clear understanding of the motives to choose between the different dosing‐regimens can facilitate more effective PrEP implementation. We therefore studied the motives for choosing for, switching between, and stopping daily or event‐driven PrEP.

**Methods:**

We used data (August 2015‐June 2017) from the prospective, longitudinal, open‐label Amsterdam PrEP demonstration study, in which daily (dPrEP) and event‐driven PrEP (edPrEP) were offered to 374 HIV‐negative MSM and two transgender persons. Participants self‐selected the preferred PrEP‐regimen at baseline and could switch regimens at three‐monthly follow‐up visits. We measured motives for choosing PrEP‐regimen at baseline and for switching and stopping PrEP at follow‐up visits. Open‐ and closed‐end items were combined and qualitatively analysed.

**Results:**

Choices of PrEP‐regimens were determined by personal and contextual factors, involving the perceived self‐efficacy concerning adherence, the risk‐context, and the anticipated impact of PrEP on physical and sexual wellbeing. dPrEP was preferred because of the anticipated better adherence and the fear of side‐effects relating to edPrEP re‐initiations. Moreover, dPrEP was perceived to be more effective than edPrEP. Motives to choose edPrEP were the expected physical burden of dPrEP, anticipated side‐effects of dPrEP, and fear to forget daily doses. Regarding the risk‐context: dPrEP was preferred for unplanned and/or frequent sex, while edPrEP was chosen when risk was predictable and/or less frequent. While some chose for dPrEP to gain more sexual freedom, others chose for edPrEP to minimize sexual risk episodes. Changes in the above factors, such as changing risk patterns, changing relationships or changing physical conditions, resulted in switching regimens. Choices to stop PrEP were related to lower sexual risk, adherence issues and side‐effects.

**Conclusions:**

The great diversity of motives illustrates the importance of offering a choice of PrEP‐regimens. In counselling of MSM starting PrEP, choices for PrEP‐regimens may be addressed as a continuum of flexible and changeable options over time. This may help individuals choose the PrEP‐regimen that best fits their current sexual context, priorities and personal capabilities and therefore will be more easily adhered to.

## 1 Introduction

The use of antiretrovirals for HIV prevention, known as pre‐exposure prophylaxis (PrEP), is proposed as one of the most promising strategies to date to further reduce HIV incidence. PrEP, the combination of tenofovir disoproxil fumarate and emitricitabine (TDF/FTC), has a high efficacy against HIV acquisition among men who have sex with men (MSM) when taken daily 1, 2, 3 and according to an event‐driven dosing schedule 4. The event‐driven PrEP‐regimen showed its efficacy among MSM in the IPERGAY trial and involves taking two TDF/FTC tablets between twenty‐four and two hours before sexual intercourse, followed by a tablet between 24 and 48 hours after the last episode of intercourse 4. Whereas daily PrEP is gradually being implemented in some European countries following the 2016 approval by the European Medicines Agency (EMA) 5 and is recommended by the WHO and other guidelines 6, event‐driven PrEP was only recently recommended for MSM in the European AIDS Clinical Society guidelines 7. Despite these developments, PrEP uptake has been slow. As for the Netherlands, a recent estimation showed that only a small proportion of PrEP‐eligible MSM actually used PrEP in 2017 8.

Event‐driven PrEP may address the challenges that appear to impede PrEP uptake and implementation, including concerns about PrEP's costs 9, 10, side‐effects, physical burden, and adhering to PrEP on a daily basis 11, 12. Event‐driven PrEP was shown to be cost‐effective 9, 10 and can pose a smaller financial burden on the health systems than daily PrEP 11, 13, 14. Furthermore, event‐driven PrEP may be more acceptable for MSM with less frequent risky sex or more concerns about PrEP's burden. Since adherence is crucial for optimal PrEP‐efficacy 1, 15, 16, MSM should be offered the choice of a PrEP‐regimen fitting their personal needs, increasing the likelihood of adherence. While there are data on the motives to use daily PrEP 17, 18, 19, little is known about the reasons to choose between daily and event‐driven PrEP, to switch between these regimens, or to discontinue PrEP use altogether in a setting that offers both regimens. The majority of previous studies reported on situations in which event‐driven PrEP was only a hypothetical option 17, 20, 21, 22, 23, 24, and where stops were associated with daily adherence problems and reduced risk 24, 25, 26. Data on actual choices between daily and event‐driven PrEP among MSM are only available from three studies in the European setting, the roll‐out programme in France 27, the Be‐Prepared study in Belgium 28, and our own study, the Amsterdam PrEP (AMPrEP) demonstration project in the Netherlands 29. Mainly quantitative data have been reported from these settings up to now related to choices between PrEP‐regimens.

In this study, we report on the motives to choose for, switch between or stop daily or event‐driven PrEP temporarily or completely in the AMPrEP demonstration study. Participants of AMPrEP were free to choose the preferred PrEP‐regimen and could freely switch between regimens at three‐monthly follow‐up visits. Understanding the motives to choose and switch between daily and event‐driven PrEP is important in order to develop better counselling options for future PrEP users that will better match their personal situation and needs. Better match between personal needs and the offered regimen should increase the overall satisfaction and adherence regarding PrEP.

## 2 Methods

### 2.1 Study design and population

AMPrEP is a prospective, longitudinal, open‐label demonstration project that aims to assess the acceptability and feasibility of offering both daily and event‐driven PrEP as part of a combination prevention package. Participants were enrolled between 3 August 2015 and 21 May 2016. Complete study procedures were published previously 29. In brief: HIV‐negative MSM and transgender persons were eligible if they were at least 18 years old and reported one or more of the following over the preceding six months: condomless anal sex (CAS) with casual partners; at least one diagnosed bacterial sexually transmitted infection (STI); use of post‐exposure prophylaxis (PEP), or an HIV‐positive partner with a detectable viral load. At baseline, after one month and subsequently on a three‐monthly basis, participants were seen for medical monitoring, counselling and data collection by self‐administered questionnaires. Participants self‐selected the PrEP‐regimen of choice at baseline and were allowed to switch PrEP‐regimens at each visit.

Ethical approval for the AMPrEP project was obtained from the ethics board of the Academic Medical Centre, Amsterdam, the Netherlands (NL49504.018.14). All AMPrEP participants provided written confirmed consent.

### 2.2 Data collection

At baseline, the motives to choose for daily or event‐driven PrEP, and reasons not to choose for the alternative regimen were collected. A member of the AMPrEP team asked participants face‐to‐face to describe in their own words the two most important underlying motives for their PrEP‐related choice(s). The answers were then directly fitted by the interviewer into predefined closed‐end answer options (which were published in an earlier format using quantitative baseline data 29) listed in Table 1. When none of the predefined items correctly reflected the participant's motive, the “other” option was chosen and the motive was quoted into an open‐text field.

**Table 1:** Data collection method, predefined categories^a^ and frequencies of the motives for choosing between daily (N = 857^a^) and event‐driven PrEP (N = 301^a^), switching between daily (N = 90^b^) and event‐driven PrEP (N = 81^b^) and stopping PrEP temporarily (N = 161^b^) and completely (N = 34^b^) in the Amsterdam PrEP study

Motives	Time point	Data collection method	Predefined categories	N (%^c^)
Choosing daily PrEP & Not choosing event‐driven PrEP	Baseline	Face‐to face interviews – fitted into predefined categories if appropriate	Because I cannot accurately guess in advance when I will be at risk for HIV infection One pill every day seems easy to me Because the schedule for intermittent PrEP seems complicated to me Because daily PrEP seems safer to me Because I want to have sex at any time without the risk of HIV infection Because I am often at risk for HIV infection Because my regular partner is HIV positive Because I would like to have sex without a condom more often Because intermittent PrEP seems less safe to me Other people insisted I should use daily PrEP *Other (open‐text field)*	233 (27.2%) 164 (19.1%) 108 (12.6%) 101 (11.8%) 52 (6.1%) 49 (5.7%) 9 (1.1%) 2 (0.2%) 0 0 *139 (16.2%)*
Choosing event‐driven PrEP & Not choosing daily PrEP	Baseline	Face‐to face interviews – fitted into predefined categories if appropriate	Because I can accurately guess in advance when I will be at risk for HIV infection I do not like taking pills every day Because I am rarely at risk for HIV infection I am afraid of the (long‐term) side effects of daily PrEP Because intermittent PrEP seems less hard on my body I am not good at taking a pill every day without forgetting Daily PrEP seems hard to keep up Because intermittent PrEP seems easier for me to do correctly I am worried that people will think that I am HIV positive *Other (open‐text field)*	87 (28.9%) 55 (18.3%) 47 (15.6%) 36 (12.0%) 13 (4.3%) 10 (3.3%) 5 (1.7%) 3 (1.0%) 0 *45 (15.0%)*
Switching to daily PrEP	3‐monthly visits	Face‐to face interviews	*Open‐text field only*	*90 (100%)*
Switching to event‐driven PrEP	3‐monthly visits	Face‐to face interviews	*Open‐text field only*	*81 (100%)*
Temporarily stopping PrEP	3‐monthly visits	Self‐administered three‐monthly questionnaire	I didn't feel like taking PrEP tablets I did not have my PrEP tablets with me I forgot I felt opposed to taking PrEP I lost my PrEP tablets *Other (open‐text field)*	25 (15.5%) 19 (11.8%) 8 (5.0%) 6 (3.3%) 1 (0.6%) *102 (63.3%)*
Completely stopping PrEP	Any time	Face‐to face interviews	*Open‐text field only*	*34 (100%)*

PrEP, pre‐exposure prophylaxis.

^a^Predefined categories were categories defined before data collection started. When none of the predefined items correctly reflected the essence of the provided personal motive during the interview, the “other” option was chosen and the motive was quoted into an open‐text field. Open‐text fields were qualitatively analysed; ^b^total number of motives collected per PrEP‐related choice; ^c^the number of times a motive was reported in pre‐defined category or open‐text field.

Similarly, at three‐monthly follow‐up visits, if relevant, participants were asked the motives for switching or stopping PrEP completely. Only open‐text fields were used to note these responses.

The motives for stopping temporarily with daily PrEP relied on self‐report using one question in the self‐administered questionnaire (“What was the reason that you temporarily stopped taking PrEP?”). Participants could self‐select a predefined item from the questionnaire or use the open‐text field.

Table 1 provides an overview of the data collection method per choice, including the frequency of reported predefined and open‐text field answers.

### 2.3 Data analyses

**Figure 1:**
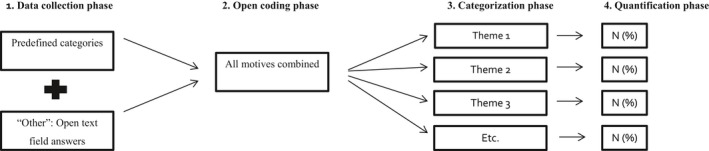
Schematic overview of the data collection and – analysis method Predefined categories (closed‐end items) were combined with open‐text field answers (“other” option). The combined list of motives were qualitatively analysed following an open‐coding process. In the categorization phase, all codes were reviewed together and crystallized into final categories, which were quantified.

Data collected between 3 August 2015 and 21 June 2017 were included in the analysis. Qualitative data analysis was performed by three researchers from different backgrounds: health sciences (HZ and SE) and psychology (UD). Figure 1 shows the data analyses process in steps. Predefined categories and open‐text fields were combined (step 1) and qualitatively analysed following an independent open‐coding process performed by two separate coders (HZ and SE) (step 2). Next, all codes were discussed and agreed upon by all three researchers and crystallized into final categories (step 3). Last, the frequencies of the final categories were determined (step 4). All qualitative analyses were performed in MAXQDA version 12.0.

### 2.4 Theoretical framework

We conducted an open explorative approach when analysing and coding our data. However, we used the Information‐Motivation‐Behavioural Skills Model (IMB) 30 and the Health Belief Model (HBM) 31 to interpret our results and to help form the recommendations for effective PrEP counselling. The IMB model was shown to be successful in the past for promoting HIV preventive behaviours 32, 33 and also formed the theoretical basis for promoting PrEP‐adherence in clinical trials 2, 34, 35, 36. Recently, the IMB model was also adapted for predicting PrEP uptake, in which social‐environmental factors such as costs or PrEP‐related stigma were recognized as moderators of PrEP uptake 37. We used the IMB model to determine the interplay between possessing sufficient information on HIV and PrEP, the individual's level of motivation to choose between PrEP‐regimens (including aspects of risk perception, attitudes and social norms), and related behavioural skills 30, 38. Further inspired by the adapted IMB model of PrEP uptake 37, we searched for social‐environmental factors that played a role in shaping the choices between daily and event‐driven PrEP. In order to further strengthen our interpretation of the motivational parts of our analysis, we included components of the Health Belief Model to our IMB approach. Our data were strongly suggestive of HBM‐like decision‐making in which the motivation to choose between regimens was based on perceived susceptibility to HIV infection and the appraisal of the balance between the costs and benefits of using each PrEP‐regimen 31.

## 3 Results

### 3.1 Descriptive

By 1 June 2016, 374 MSM and 2 TGSM had enrolled and started PrEP. Their median age was 39.5 years (interquartile range (IQR), 32 to 48). The majority were Caucasian (85.1%) and highly educated (76.1%).

At start, 273 participants chose daily PrEP and 103 chose event‐driven PrEP. During follow‐up, 86.4% of participants had at least one visit in the past three months. Participants switched 53 times to event‐driven PrEP and 56 times to daily PrEP. Daily PrEP was temporarily stopped 161 times. Twenty‐one participants stopped completely with PrEP.

Table 2 provides an overview of the motives to choose and switch between daily and event‐driven PrEP, and Table 3 of the motives to temporarily or completely stop PrEP. All tables include the frequency at which motives were reported, their proportions relative to all reported motives by all participants, and representative quotations. Below, we explain a more in‐depth selection of key findings from the tables.

**Table 2:** Motives to choose at baseline and switch at follow‐up between daily and event‐driven PrEP, frequency of motives, and representative quotes^a^ among 376 MSM & TGSM, Amsterdam, 2015 to 2017

Thematic Continuums	Motives to choose daily PrEP (N = 857) among 273 participants^b^	n (%)^c^		Motives to switch to daily PrEP (N = 90) among 56 participants^b^	n (%)^c^		Motives to choose event‐driven PrEP (N = 301) among 103 participants^b^	n (%)^c^ n		Motives to switch to event‐driven PrEP (N = 81) among 53 participants^b^	n (%)^c^
1. Perceived HIV risk	Sex is unpredictable/frequent/risky	357 (41.7)		Sex became unpredictable/frequent/risky	21 (23.3)		Sex is predictable/infrequent/low‐risk	145 (48.1)		Sex became predictable/infrequent/low‐risk	22 (27.2)
2.1A	“I cannot plan sex”		2.1C	“I had more sex than expected, so daily PrEP seems better to me”		2.1F	“Because I would have to take a pill every day while I am not at risk/have no sex”		2.1H	“(…) now a monogamous relationship, so no risk for HIV”	
2.1B	“[I] have a lot of changing contacts, sometimes unsafe”		2.1D	[I] already often used PrEP on a daily basis”		2.1G	“I am dependent on sex parties as I usually cannot get sex, thus sex is planned”		2.1J	“[Viral load of positive] partner remains undetectable due to monotherapy provided in a clinical trial”	
			2.1E	“Steady partner is going to use new cART regime with a chance of blips”							
2. Adherence considerations	Expecting issues with event‐driven PrEP adherence	349 (40.7)		Experiencing issues with event‐driven PrEP adherence	25 (27.7)		Expecting Issues with daily PrEP adherence	20 (6.6)		Experiencing issues with daily PrEP adherence	27 (33.3)
2.2A	“[Dosing] every day is easier. Otherwise [I might] possibly forget [event‐driven PrEP]”		2.2C	“I don't trust myself with event‐driven PrEP, [I have] had PEP in between”		2.2E	“[I am] afraid I might forget a pill when using it on a daily basis”		2.2F	“[I] used to forget PrEP sometimes, felt guilty afterwards”	
2.2B	“More routine and structure. It [daily PrEP scheme] is less complicated, will probably increase adherence”		2.2D	“[I] prefer more structure, no different time points”					2.2G	“Daily pill‐taking causes stress”	
3. Perceived safety, efficacy and burden of the regimen	Higher perceived efficacy and safety of daily PrEP	118 (13.8)		Higher perceived efficacy and safety of daily PrEP	12 (13.3)		Toxicity and burden of daily medication	97 (3.2)			0
2.3A	“[I] think that it will be more effective because I will use it daily; constant drug level in the body”		2.3C	“[I am] not convinced of the effectiveness of the intermittent scheme		2.3D	“It is still medication, [I] don't want to take it every day”				
2.3B	“Because I can become resistant”										
4. Anticipated or experienced side‐effects	Fear of recurring side‐effects with event‐driven PrEP	6 (<1)		Experiencing recurring side‐effects with event‐driven PrEP	9 (10.0)		Fear of continuous side‐effects with daily PrEP	36 (1.2)		Experiencing continuous side‐effects with daily PrEP	21 (25.9)
2.4A	“[Participant] thinks that side‐effects will reoccur every time”		2.4B	“Taking two pills at once gives a high feeling; prefers daily”		2.4C	“I am afraid of the (long‐term) side‐effects of daily PrEP”		2.4D	“[Participant] feels like daily PrEP has too many constant side‐effects”	
5. Freedom versus control over sexual behaviour	To maintain/gain more sexual freedom	8 (<1)		To maintain/gain more sexual freedom	22 (24.4)		To inhibit/control sexual risk episodes	1 (<1)		To inhibit/control sexual risk episodes	3 (3.7)
2.5A	“Because I can forget about HIV completely, I do not have to think about it anymore”		2.5B	“It is hard to predict when he will have sex. [He] wants to be able to have sex immediately.”		2.5C	“Because event‐driven PrEP makes you think [about your sex life], and then you don't go all crazy”		2.5D	“Event‐driven PrEP is considered as a big stick to do it safe”	
6. Experimenting with the regimen	Experimenting with the daily PrEP‐regimen	12 (1.4)		Experimenting with the daily regimen	1 (1.1)		Experimenting with the event‐driven PrEP‐regimen	1 (<1)		Experimenting with the daily regimen	1 (1.2)
2.6A	“First see how body reacts on continuous intake”		2.6B	“[I] want to know what it is like to take it [PrEP] daily. How will it influence my behaviour?”		2.6C	“Try to see if this is the right schedule, first experience [it]”		2.6D	“[I] would like to see if the event‐driven schedule is feasible for me”	
7. Other	Daily PrEP provides solidarity with daily medication users	7 (<1)			0		Event‐driven PrEP is cheaper	1 (<1)			0
2.7A	“Would like to take the pill together with steady partner, he is HIV‐positive”					2.7B	“Event‐driven PrEP is cheaper”				

cART, combination antiretroviral therapy; MSM, men who have sex with men; PrEP, pre‐exposure prophylaxis; TGSM, transgender persons who have sex with men.

^a^Quotes were originally in Dutch. Dutch quotes have been translated into English; ^b^Since participants reported at least one reason to choose and switch between PrEP‐regimens, the number of motives analysed is larger than the number of participants; ^c^the number of times a specific motive was reported, not equal to the number of participants. Percentages reflect the proportion of all motives reported.

**Table 3:** Motives for temporarily or completely stopping PrEP, frequency of motives, and representative quotations MSM & TGSM temporarily or completely stopping PrEP at follow‐up, Amsterdam, 2015 to 2017

	n (%)^b^	Quote nr.	Representative quotes^c^
Motives for temporarily stopping daily PrEP use (>3 days) (N = 161) among 95 participants^a^			
1. Adherence and aversion issues	71 (44.1)	3.10A	“Lost them on holiday”
2. Temporary reduction in risk circumstances	51 (31.7)	3.11A	“I was in the countryside where there are no gays, so also no chances on having sex”
		3.11B	“I had an STI so I was not allowed to have sex”
		3.11C	“Temporarily had no sex besides steady partner”
		3.11D	“[I used] the pills around sexual episodes”
3. Sickness or poor health conditions	30 (18.6)	3.12A	“[I] needed surgery and was advised to stop briefly”
		3.12B	“[I] was not feeling well so [I] stopped with PrEP until I recovered”
4. Side‐effects and physical burden issues	8 (5.0)	3.13A	“[I] had other daily medication, [I] did not want to burden myself too much”
5. Wanting to control sexual risk behaviour	1 (<1)	3.14A	“Stop [with daily PrEP] in order to have solely protected sex”
Motives for stopping PrEP use completely (N = 34) among 21 participants^d^			
1. No more need for PrEP	20 (58.8)	3.15A	“[I am] not at risk of HIV anymore”
		3.15B	“The idea that you take medication for something you can also use condoms for”
2. Unacceptable side‐effects	10 (29.4)	3.16A	“Participant wasn't able to enjoy sex due to side‐effects”
3. Other	2 (5.9)	3.17A	“Obliged to stop due to disability insurance for self‐employed persons, otherwise not insurable”
		3.17B	“He notices that he sought more extremes due to several circumstances, PrEP was one of them”
4. Aversion against daily medication	1 (2.9)	3.18A	“Aversion against daily medication”
5. Dissatisfaction with study procedures	1 (2.9)	3.19A	“The too‐long questionnaires”

MSM, men who have sex with men; PrEP, pre‐exposure prophylaxis; STI, sexually transmitted infection; TGSM, transgender persons who have sex with men.

^a^Participants reported only one reason to temporarily stop PrEP. The number of motives analysed is therefore equal to the total number of participants that temporarily stopped PrEP; ^b^the number of times a specific motive was reported, not equal to the number of participants. Percentages reflect the proportion of all motives (N = 658) reported; ^c^quotes were originally in Dutch and have been translated into English; ^d^since participants reported at least one reason to stop PrEP, the number of motives analysed is larger than the number of participants.

### 3.2 Motives for choosing between daily and event‐driven PrEP

Seven motivational themes were distinguished to describe the choices made between PrEP‐regimens, based on 1329 recorded reasons among 376 participants for choosing one regimen over the other at baseline or during follow‐up (Table 2). In most cases, the motives to use the daily or event‐driven regimen shared the same overarching themes but on different ends of their spectra, and were therefore categorized as thematic continuums.

The *predictability and frequency of risky sex acts* were predominant motives to choose between PrEP‐regimens. Daily PrEP was chosen by participants whose desire to maintain sexual spontaneity made them unwilling or unable to plan their sex acts (quote 2.1A). Also, participants who often have condomless sex or frequently change sex partners opted for daily PrEP (quote 2.1B). Event‐driven PrEP was favoured by participants who reported infrequent risk (quote 2.1F) or predictable risk (i.e. having planned sex only or sex within specific circumstances) (quote 2.1G).

A large proportion of participants took their *expected adherence* into account. Participants who chose daily PrEP were afraid to forget or make mistakes with PrEP intake if using it on an event‐driven basis (quote 2.2A, 2.2B). On the other end of the spectrum, some participants chose event‐driven PrEP because they questioned their ability to adhere to daily medication correctly (quote 2.2E).

Other motives that guided the choices made between regimens were participants’ beliefs about the *expected safety, efficacy, and burden of the regimens* and *fear of side‐effects*. Some participants perceived the preventive efficacy of daily PrEP to be higher because they expected, for example, a more stable drug level in their blood (quote 2.3A). Some perceived a smaller chance of recurring side‐effects (quote 2.4A) and resistance development (quote 2.3B). However, some participants choosing event‐driven PrEP disliked daily medication for its toxicity and expected physical burden (quote 2.4C) and therefore wanted to take as little unnecessary medication as possible (quote 2.3D).

Choice of regimen also depended on its expected impact on the *freedom in‐ or control over sexual behaviour*. On the one hand, daily PrEP was associated with the ability to have condomless sex and reduce fear of HIV during sex (quote 2.5A). On the other hand, event‐driven PrEP was seen as a way to control or limit one's own sexual risk episodes (quote 2.5C).

### 3.3 Motives for switching between PrEP‐regimens

We identified six motivational themes related to switching between PrEP‐regimens (Table 2). They predominantly reflected changing risk circumstances or negative experiences with the regimen previously chosen.

The majority of switching participants *experienced difficulties with the uptake practices* of the regimen they initially chose. Some participants made mistakes with the event‐driven PrEP schedule, which caused stress and anxiety (quote 2.2C). Others switched to daily PrEP for the structure it was believed to provide (quote 2.2D). In contrast, some participants had difficulties adhering properly to daily medication and therefore switched to event‐driven PrEP (quote 2.2F). Others developed an aversion to taking daily medication (quote 2.2G) or to taking more medication than needed.

*Changing HIV infection risk* was another frequent motive for switching regimens. Participants who had more sex partners (than initially anticipated) or expected an increase, switched to daily PrEP (quote 2.1C). Daily PrEP was also chosen by those who ended monogamous relationships or who increasingly engaged with HIV‐positive sex partners. Some participants noted that the frequency of their event‐driven PrEP intake was so high that they might as well use PrEP daily (quote 2.1D). On the other hand, participants who had less risky sex (than initially anticipated) switched to event‐driven PrEP. For example, some participants stopped having sex with multiple partners because of relationship problems or entering monogamous relationships (quote 2.1H). Other participants saw event‐driven PrEP as a better match for their current risk pattern, for example, sex limited to weekends only. Anticipated alterations in the viral load of HIV‐positive partners were also motives to switch between regimens, as they changed participants’ perceived HIV‐infection risk (quote 2.1E, 2.1J).

Other motives for switching included the *fear or experience of side‐effects* attributable to PrEP. Users of event‐driven PrEP who experienced side‐effects at each dose of PrEP hoped that daily PrEP would improve this situation (quote 2.4B). On the other hand, daily users who had continual side‐effects hoped that event‐driven dosage would bring relief (quote 2.4D).

*Gaining versus limiting freedom in sexual behaviour* remained an important thematic continuum when switching between regimens. Some participants were motivated to switch to daily PrEP by a desire for more freedom and spontaneity in sex and its planning (quote 2.5B), or because they realized they were able to plan their sexual activities. On the other hand, switching to event‐driven PrEP was seen as a strategy to reduce sexual risk‐taking and enhance desired condom use (quote 2.5D). For other participants, event‐driven PrEP seemed more feasible as they became more able to plan sexual activities, or desired to do so.

### 3.4 Motives for temporarily and completely stopping PrEP

We distinguished five motivational themes for the 161 reasons among 95 participants to temporarily stop daily PrEP and five motivational themes for the 43 reasons among 21 participants to completely stop using PrEP. Temporary and complete PrEP stops were most commonly based on adherence and aversion issues (quotes 3.10A, 3.18A); a perception of temporary reduced need for PrEP, for example, because of traveling (quote 3.11A) or a perception of permanently reduced HIV‐risk (quote 3.15A); periods of sickness (quotes 3.12A, 3.12B), and the experience and unacceptability of side‐effects (quote 3.16A). Further motives to completely stop with PrEP were personal insurance issues (quote 3.17A), and feeling like PrEP provoked undesired extreme risk behaviour (quote 3.17B).

## 4 Discussion

In this study, we gained detailed insights into the motives and rationales behind choices between daily and event‐driven PrEP‐regimens and why such choices change over time. We found that a great variety of individual and contextual factors determine the choices for starting PrEP and choosing between PrEP‐regimens at initiation. Changes in such factors, or growing experience with initial motives during follow‐up, resulted in switching PrEP‐regimens and stopping PrEP temporarily or completely.

To the best of our knowledge, this is the first qualitative study to report on the motives for choosing and switching between daily and event‐driven PrEP‐regimens. We showed that switching occurred on a fairly large scale, and that both choosing a regimen and switching between regimens were based on a variety of expectations and experiences regarding the physical, sexual and psychosocial impact of the use of each regimen. Some participants even chose to try out each regimen to experience its physical and behavioural effect before committing to one. In addition, earlier quantitative analyses from the AMPrEP showed that older age and being involved in a steady relationship were associated with choosing event‐driven PrEP and that daily medication use and more CAS episodes in the preceding months were associated with choosing daily PrEP 29. The above results highlight the added value of offering a choice between PrEP‐regimens, as it enables MSM to match and adjust HIV prevention strategies to their potentially changeable individual needs and the sexual context of their life. If individual needs are not met, adherence can decline 15, 16, 39, 40, 41, 42, 43, 44, 45, 46, 47, 48. The adjustability of PrEP‐regimens to changing life circumstances contributes to a personalized HIV prevention approach that potentially can maximize PrEP's potential as a public health measure.

General motives to temporarily or completely stop PrEP were in line with expectations, problems with uptake such as travelling, sickness and side‐effects, as noted in previous studies 39, 43, 44, 46, 48. The motives to temporarily stop daily PrEP or switch to event‐driven PrEP were primarily related to decreases in sexual risk behaviour. Our data therefore provide real‐life support to earlier data on intentions to use or stop PrEP, showing that PrEP users temporarily stopped daily PrEP as they found themselves in a non‐risky episode of their lives, a phenomenon referred to as “seasons of risk” 21, 44, “seasons of vulnerability” 49 and “seasons of PrEP ”^50^. While some studies conceptualized such episodes of reduced intake as indication of non‐adherence to the daily regimen 15, 16, 40, 43, 44, we argue, among other authors 24, 42, 51, 52, 53, that such well‐informed decisions indicate self‐efficacy in managing PrEP‐regimens according to current risk level. In some cases, it might be indicative of an informal and temporary switch to an event‐driven regimen. We therefore suggest that all PrEP users should be educated about safe ways of stopping and re‐starting PrEP, even when on the daily regimen. Healthcare providers should evaluate whether such choices correspond to actual reduced risk and advice men accordingly. The observed discordance between objective and subjective HIV risk among potential PrEP users 54 and documented cases who acquired HIV after stopping PrEP 3, 4, 55, 56, 57 show that provider training must incorporate early and extensive counselling on alternative risk‐reduction strategies when stopping PrEP.

For a minority of participants, switching to event‐driven PrEP and temporarily or completely stopping PrEP were motivated by the need to control the frequency and level of their sexual risk behaviour. Such motives suggest that a few of our participants looked for options to manage what they perceived to be undesired behaviour resulting from PrEP use, such as decreasing condom use or feeling less in control sexually. Event‐driven PrEP was seen as a strategy that enabled more control over sexual risk behaviour compared to daily PrEP.

Based on the principles of the IMB 30 and HBM models 31, our data suggest that a PrEP‐regimen is chosen based on participant's knowledge of HIV‐related risk as well as on knowledge of the effectiveness and safety of PrEP‐regimens. Expectations regarding the perceived physical burden in terms of toxicity and side‐effects, and the perceived impact a regimen can have on their sex life guided the motivation to choose between the regimens. Some motivational aspects of regimen‐choices strongly point towards HBM‐type decision‐making in which risk perceptions and expected benefits of using a certain PrEP regimen are weighted against the costs of the alternative regimen. The changes in perceived HIV‐risk was the predominant reason to switch between – or stop with a PrEP‐regimen. In addition, the perceived or experienced behavioural skills to adhere to a specific regimen co‐determined the choice between regimens. Hence, most reasons for switching and stopping PrEP indicate a re‐evaluation of the initial cost/benefit evaluations, leading to reconsideration of the appropriate regimen match. While our theoretical framework was open for the exploration of environmental factors such as social, structural and normative factors, our data strongly suggest that the factors playing a role in choosing or switching a PrEP regimen were individual‐based. This is probably due to AMPrEP being a demonstration project where costs and other structural barriers played no role. Furthermore, social‐environmental factors such as stigma, that are known to play a role in the motivation to start 24, 58 or temporarily stop PrEP use 59, may play less of a role once the decision to start PrEP was already taken.

We suggest that IMB and HBM principles can be used to develop PrEP counselling targeted at (future) PrEP users to explore the best match between individual needs and capabilities and PrEP regimen characteristics. Changes in the cost‐benefit balance of using a specific PrEP‐regimen in relation to changes in risk behaviour or evolving self‐efficacy issues regarding adherence can be explored during follow‐up visits to facilitate decision‐making about switching regimens, or whenever changes require re‐evaluation of PrEP as the right HIV protection method.

Limitations of our findings are that our study population constitutes a highly‐educated sample of early adopters with very few transgender persons involved 29, similar to other PrEP studies 3, 4, 40, 60. Our project may therefore not be representative of the wider PrEP‐eligible population in the Netherlands. In addition, considering our participants are all part of a demonstration trial, further research should explore social‐environmental and structural motives and barriers that might exist in real‐world settings that can influence the choices between using daily and event‐driven PrEP regimens. Finally, we rely on participants’ self‐report of switches and stops at each follow‐up visit and cannot rule out interim switches or intentional misreport of used regimens for personal benefits.

## 5 Conclusions

Our findings underscore the importance of offering a choice of PrEP‐regimens to match user's priorities and needs for HIV prevention. Choices between PrEP‐regimens are based on many motives, involving the changing risk‐context, perceived and actual self‐efficacy concerning adherence, and the anticipated or experienced impact of PrEP on physical and sexual wellbeing. To facilitate future PrEP use, enhance adherence and safeguard PrEP‐efficacy, counselling that incorporates considerations towards the individual motives behind choices of PrEP‐regimens could help individuals choose their future PrEP‐regimen in a way that best matches their sexual context, priorities and capabilities. For PrEP implementation to be successful and have an optimal impact on the epidemic, we believe a tailored approach is needed in which the choices for PrEP‐regimens are addressed as a continuum of flexible and changeable options over time.

## Competing interests

Our institute received the study drug for the Amsterdam PrEP study from Gilead Sciences. EH received financial reimbursement for time spent serving on advisory boards of Gilead Sciences, and a speaker fee from Janssen‐Cilag, paid to her institute. MP obtained unrestricted research grants and speaker's fees from Gilead Sciences, Roche, Abbvie, and MSD, paid to her institute. UD obtained unrestricted research grants and speaker's fees from Gilead Sciences paid to the institute only. The remaining authors declared no potential conflicts of interests for this project.

## Authors' contributions

EH, RA, MP, HdV, MSvdL and UD contributed to study concept and design. HZ, SE, RA, EH and UD contributed to acquisition, analysis or interpretation of the data. HZ, SE and UD performed all descriptive and qualitative analyses. HZ drafted the manuscript under supervision of all co‐authors. All authors critically revised and approved the final version for publication.

## Supporting information


**Appendix S1.** Acknowledgements of the H‐TEAM consortium.
