# Supplementary material for: Motives for choosing, switching and stopping daily or event‐driven pre‐exposure prophylaxis – a qualitative analysis
Source: J Int AIDS Soc. 2019 Oct 14;22(10):e25389. doi: 10.1002/jia2.25389 (PMC6791997; doi:10.1002/jia2.25389)
Supplement: Supplementary file 1 — Appendix S1. Acknowledgements of the H‐TEAM consortium. [file JIA2-22-e25389-s001.docx]

# Appendix S1: Acknowledgements of the H-TEAM consortium

H-TEAM Steering Committee: J.E.A.M. van Bergen^6;4;5^, G.J. de Bree^1;2^, P. Brokx^8^, F. Deug^6^, M. Heidenrijk^1^, M. Prins^3;2^, P. Reiss^1;7^ (chair), M. van der Valk^2^

H-TEAM Core Project Group: J.E.A.M. van Bergen^6;4;5^, G.J. de Bree^1;2^ (chair), P. Brokx^8^, U. Davidovich^3^, S.E. Geerlings^2^, E. Hoornenborg^3^, A. Oomen^6^, A. van Sighem^7^, W. Zuilhof^6^ H-TEAM

Project Management: M.L. Groot Bruinderink^1^

H-TEAM additional collaborators:

R.C.A. Achterbergh^3^, M. van Agtmael^24^, J. Ananworanich^22^, D. Van de Beek^17^, G.E.L. van den Berk^11^, D. Bezemer^7^, A. van Bijnen^6^, W.L. Blok^11^, S. Bogers^2^, M. Bomers^24^, C.A.B. Boucher^13^, W. Brokking^26^, D. Burger^20^, K. Brinkman^11^, N. Brinkman^32^, M. de Bruin^12^, S. Bruisten^3^, L. Coyer^3^, R. van Crevel^29^, C.G. Daans^3;34^, L. Dellemann^6^, M. Dijkstra^3^, Y.T. van Duijnhoven^3^, A. van Eeden^26^, L. Elsenburg^26^, M.A.M. van den Elshout^3^, C. Ester^7^, E. Ersan^3^, P. E.V. Felipa^3^, P.H.J. Frissen^11^, T.B.H. Geijtenbeek^18^, M.H. Godfried^2^, J. van Gool^3^, A. Goorhuis^2^, M. Groot^26^, C.A. Hankins^1^, A. Heijnen^30;31^, M.M.J Hillebregt^7^, A. Hogewoning^3^, M. Hommenga^3^, J.W. Hovius^2^, Y. Janssen^32^, K. de Jong^3^, V. Jongen^3^, N.A. Kootstra^19^, R.A. Koup^21^, F.P. Kroon^16^, T.J.W. van de Laar^35;36^, F. Lauw^37^, M. M. van Leeuwen^3^, K. Lettinga^27^, I. Linde^3^, D.S.E. Loomans^3^, J.T. van der Meer^2^, , T. Mouhebati^6^, B.J. Mulder^3^, J. Mulder^25^, F.J. Nellen^2^, A. Nijsters^6^, H. Nobel^2^, P. Oostvogel^3^, E.L.M. Op de Coul^5^, E. Peters^24^, I.S. Peters^3^, T. van der Poll^2^, O. Ratmann^28^, C. Rokx^14^, M.S. van Rooijen^3^, M.F. Schim van der Loeff^3;10^, W.E.M. Schoute^11^, G.J. Sonder^3^, J. Veenstra^27^, A. Verbon^14^, F. Verdult^8^, J. de Vocht^24^, H.J. de Vries^3;9;10^, S. Vrouenraets^27^, M. van Vugt^2^, W.J. Wiersinga^2^, F.W. Wit^2;7^, L.R. Woittiez^2^, S. Zaheri^7^, P. Zantkuijl^6^, M.C. van Zelm^23^, A. Żakowicz^33^, H.M.L. Zimmermann^3^.

1 Department of Global Health, Amsterdam UMC – location AMC, and Amsterdam Institute for Global Health and Development, Amsterdam, the Netherlands

2 Department of Internal Medicine, Division of Infectious Diseases, Amsterdam UMC – location AMC, Amsterdam, the Netherlands

3 Department of Infectious Diseases, Public Health Service of Amsterdam, Amsterdam, the Netherlands

4 Department of General Practice, Amsterdam UMC – location AMC, University of Amsterdam, Amsterdam, the Netherlands

5 Epidemiology and Surveillance Unit, Center for Infectious Disease Control, National Institute of Public Health and the Environment, the Netherlands

6 STI AIDS Netherlands, Amsterdam, the Netherlands

7 Stichting HIV Monitoring, Amsterdam, the Netherlands

8 Dutch Association of PLHIV, Amsterdam, the Netherlands

9 Department of Dermatology, Amsterdam UMC – location AMC, University of Amsterdam, Amsterdam, the Netherlands

10 Center for Infection and Immunology, Amsterdam (CINIMA), Amsterdam UMC – location AMC, University of Amsterdam, Amsterdam, the Netherlands

11 Department of internal medicine, OLVG – location East, Amsterdam, the Netherlands

12 Aberdeen Health Psychology Group, Institute of Applied Health Sciences, University of Aberdeen, Aberdeen, United Kingdom

13 Department of viro-science, Erasmus Medical Center Rotterdam, Rotterdam, the Netherlands

14 Department of Internal Medicine and Infectious Diseases, Erasmus Medical Center, Rotterdam, the Netherlands

16 Department of Infectious Diseases, Leiden University Medical Center, Leiden, the Netherlands

17 Center of Infection and Immunity Amsterdam (CINIMA), Department of Neurology, Amsterdam UMC – location AMC, Amsterdam, the Netherlands

18 Laboratory of Experimental Immunology, Amsterdam UMC – location AMC Amsterdam, the Netherlands

19 Laboratory for Viral Immune Pathogenesis, Amsterdam UMC – location AMC Amsterdam, the Netherlands

20 Department of Pharmacy, Radboud University Nijmegen Medical Center, Nijmegen, the Netherlands

21 Immunology Laboratory, Vaccine Research Center, NIAID, National Institutes of Health

22 US Military HIV Research Program and the Henry M. Jackson Foundation for the Advancement of Military Medicine, Bethesda, United States

23 Department of Virology, Erasmus Medical Center, Rotterdam, the Netherlands

24 Department of Internal Medicine, Amsterdam UMC – location VUMC, Amsterdam, the Netherlands

25 Department of Internal Medicine, Slotervaart Hospital, Amsterdam, the Netherlands

26 DC Clinics, Amsterdam, the Netherlands

27 Department of Internal Medicine, OLVG – location West , Amsterdam, the Netherlands

28 School of Public Health, Faculty of Medicine, Imperial College London, London, United Kingdom

29 Department of Internal Medicine, Radboud University Nijmegen Medical Center, Nijmegen, the Netherlands

30 Sexology Center Amsterdam, Amsterdam, the Netherlands

31 GP practice Heijnen & de Meij, Amsterdam, the Netherlands

^32^ Elaa – First line Amsterdam Almere, Amsterdam, the Netherlands

^33^ AIDS Healthcare Foundation, Amsterdam, the Netherlands

^34^ Center of Expertise on Gender Dysphoria, Amsterdam UMC – location VUMC, Amsterdam, the Netherlands

^35^ Department of Medical Microbiology, OLVG, Amsterdam, the Netherlands

^36^ Department of Donor Medicine Research, Laboratory of Blood-borne Infections, Sanquin Research, Amsterdam, the Netherlands

^37^ Department of Internal Medicine, Medical Center Jan van Goyen, Amsterdam, the Netherlands

| **Table 1. Data collection method, predefined categories**^†^ **and frequencies of the motives for choosing between daily (N=857**^†^**) and event-driven PrEP (N=301**^†^**), switching between daily (N=90**^‡^**) and event-driven PrEP (N=81**^‡^**) and stopping PrEP temporarily (N=161**^‡^**) and completely (N=34**^‡^**) in the Amsterdam PrEP study** | | | | |
| --- | --- | --- | --- | --- |
| **Motives** | **Time point** | **Data collection method** | **Predefined categories** | **N (%**^§^**)** |
| **Choosing daily PrEP & Not choosing event-driven PrEP** | Baseline | Face-to-face interviews – fitted into predefined categories if appropriate | Because I cannot accurately guess in advance when I will be at risk for HIV infection  One pill every day seems easy to me  Because the schedule for intermittent PrEP seems complicated to me  Because daily PrEP seems safer to me  Because I want to have sex at any time without the risk of HIV infection  Because I am often at risk for HIV infection  Because my regular partner is HIV positive  Because I would like to have sex without a condom more often  Because intermittent PrEP seems less safe to me  Other people insisted I should use daily PrEP  *Other (open-text field)* | 233 (27.2%)  164 (19.1%)  108 (12.6%)  101 (11.8%)  52 (6.1%)  49 (5.7%)  9 (1.1%)  2 (0.2%)  0  0  *139 (16.2%)* |
| **Choosing event-driven PrEP & Not choosing daily PrEP** | Baseline | Face-to-face interviews – fitted into predefined categories if appropriate | Because I can accurately guess in advance when I will be at risk for HIV infection  I do not like taking pills every day  Because I am rarely at risk for HIV infection  I am afraid of the (long-term) side effects of daily PrEP  Because intermittent PrEP seems less hard on my body  I am not good at taking a pill every day without forgetting  Daily PrEP seems hard to keep up  Because intermittent PrEP seems easier for me to do correctly  I am worried that people will think that I am HIV positive  *Other (open-text field)* | 87 (28.9%)  55 (18.3%)  47 (15.6%)  36 (12.0%)  13 (4.3%)  10 (3.3%)  5 (1.7%)  3 (1.0%)  0  *45 (15.0%)* |
| **Switching to daily PrEP** | 3-monthly visits | Face-to-face interviews | *Open-text field only* | *90 (100%)* |
| **Switching to event-driven PrEP** | 3-monthly visits | Face-to-face interviews | *Open-text field only* | *81 (100%)* |

| **Table 1.** *(Continued)* | | | | |
| --- | --- | --- | --- | --- |
| **Motives** | **Time point** | **Data collection method** | **Predefined categories** | **N (%**^‡^**)** |
| **Temporarily stopping PrEP** | 3-monthly visits | Self-administered three-monthly questionnaire | I didn’t feel like taking PrEP tablets  I did not have my PrEP tablets with me  I forgot  I felt opposed to taking PrEP  I lost my PrEP tablets  *Other (open-text field)* | 25 (15.5%)  19 (11.8%)  8 (5.0%)  6 (3.3%)  1 (0.6%)  *102 (63.3%)* |
| **Completely stopping PrEP** | Any time | Face-to-face interviews | *Open-text field only* | *34 (100%)* |

PrEP = pre-exposure prophylaxis.

^†^Predefined categories were categories defined before data collection started. When none of the predefined items correctly reflected the essence of the provided personal motive during the interview, the “other” option was chosen and the motive was quoted into an open-text field. Open-text fields were qualitatively analysed.

^‡^Total number of motives collected per PrEP-related choice.

^§^The number of times a motive was reported in predefined category or open-text field.

| **Table 2. Motives to choose at baseline and switch at follow-up between daily and event-driven PrEP, frequency of motives, and representative quotes**^†^ **among 376 MSM & TGSM, Amsterdam, 2015-2017** | | | | | | | | | | | |
| --- | --- | --- | --- | --- | --- | --- | --- | --- | --- | --- | --- |
| **Thematic Continuums** | **Motives to choose**  **daily PrEP (N=857) among 273 participants^‡^** | **n (%)**^§^ |  | **Motives to switch to daily PrEP**  **(N=90) among 56 participants**^‡^ | **n**  **(%)**^§^ |  | **Motives to choose**  **event-driven PrEP (N=301) among 103 participants^‡^** | **n**  **(%)**^§^ **n** |  | **Motives to switch to**  **event-driven PrEP**  **(N=81) among 53 participants^‡^** | **n (%)^§^** |
| **1. Perceived**  **HIV risk** | Sex is unpredictable/ frequent/risky | 357 (41.7) |  | Sex became unpredictable/  frequent/risky | 21 (23.3) |  | Sex is predictable/  infrequent/low-risk | 145 (48.1) |  | Sex became predictable/infrequent/low-risk | 22 (27.2) |
| *2.1A* | *”I cannot plan sex”* |  | *2.1C* | *“I had more sex than expected, so daily PrEP seems better to me”* |  | *2.1F* | *“Because I would have to take a pill every day while I am not at risk/have no sex”* |  | *2.1H* | *“(…) now a monogamous relationship, so no risk for HIV”* |  |
| *2.1B* | *“[I] have a lot of changing contacts, sometimes unsafe”* |  | *2.1D* | *[I ] already often used PrEP on a daily basis”* |  | *2.1G* | *“I am dependent on sex parties as I usually cannot get sex, thus sex is planned”* |  | *2.1*  *J* | *“[Viral load of positive] partner remains undetectable due to monotherapy provided in a clinical trial”* |  |
|  |  |  | *2.1*  *E* | *“Steady partner is going to use new cART regime with a chance of blips”* |  |  |  |  |  |  |  |
| **2. Adherence considerations** | Expecting issues with event-driven PrEP adherence | 349 (40.7) |  | Experiencing issues with event-driven PrEP adherence | 25 (27.7) |  | Expecting Issues with daily PrEP adherence | 20 (6.6) |  | Experiencing issues with daily PrEP adherence | 27 (33.3) |
| *2.2A* | *“[Dosing] every day is easier. Otherwise [I might] possibly forget [event-driven PrEP]”* |  | *2.2C* | *“I don’t trust myself with event-driven PrEP, [I have] had PEP in between”* |  | *2.2E* | *“[I am] afraid I might forget a pill when using it on a daily basis”* |  | *2.2*  *F* | *“[I] used to forget PrEP sometimes, felt guilty afterwards”* |  |
| *2.2B* | *“More routine and structure. It [daily PrEP scheme] is less complicated, will probably increase adherence”* |  | *2.2D* | *“[I] prefer more structure, no different time points”* |  |  |  |  | *2.2G* | *“Daily pill-taking causes stress”* |  |
| **3. Perceived safety, efficacy and burden of the regimen** | Higher perceived efficacy and safety of daily PrEP | 118 (13.8) |  | Higher perceived efficacy and safety of daily PrEP | 12 (13.3) |  | Toxicity and burden of daily medication | 97 (3.2) |  |  | 0 |
| *2.3A* | *“[I] think that it will be more effective because I will use it daily; constant drug level in*  *the body”* |  | *2.3C* | *“[I am] not convinced of the effectiveness of the intermittent scheme* |  | *2.3D* | *“It is still medication, [I] don’t want to take it every day”* |  |  |  |  |
| *2.3B* | *“Because I can become resistant”* |  |  |  |  |  |  |  |  |  |  |

| **Table 2.** *(Continued)* | | | | | | | | | | | |  |
| --- | --- | --- | --- | --- | --- | --- | --- | --- | --- | --- | --- | --- |
| **Thematic Continuums** | **Motives to choose**  **daily PrEP (N=857) among 273 participants^‡^** | **n (%)**^§^ |  | **Motives to switch to daily PrEP (N=90) among 56 participants^‡^** | **n (%)**^§^ |  | **Motives to choose**  **event-driven PrEP (N=301) among 103 participants^‡^** | **n (%)**^§^ **n** |  | **Motives to switch to**  **event-driven PrEP (N=81) among 53 participants^‡^** | **n (%)^§^** | |
| **4. Anticipated or experienced side-effects** | Fear of recurring side-effects with event-driven PrEP | 6  (<1) |  | Experiencing recurring side-effects with event-driven PrEP | 9 (10.0) |  | Fear of continuous side-effects with daily PrEP | 36 (1.2) |  | Experiencing continuous side-effects with daily PrEP | 21 (25.9) | |
| *2.4A* | *“[Participant] thinks that side-effects will reoccur every time”* |  | *2.4B* | *“Taking two pills at once gives a high feeling; prefers daily”* |  | *2.4C* | *“I am afraid of the (long-term) side-effects of daily PrEP”* |  | *2.4D* | *“[Participant] feels like daily PrEP has too many constant side-effects”* |  | |
| **5. Freedom versus control over sexual behaviour** | To maintain/gain more sexual freedom | 8  (<1) |  | To maintain/gain more sexual freedom | 22 (24.4) |  | To inhibit/control sexual risk episodes | 1  (<1) |  | To inhibit/control sexual risk episodes | 3 (3.7) | |
| 2.5A | *“Because I can forget about HIV completely, I do not have to think about it anymore”* |  | *2.5B* | *“It is hard to predict when he will have sex. [He] wants to be able to have sex immediately.”* |  | *2.5C* | *“Because event-driven PrEP makes you think [about your sex life], and then you don’t go all crazy”* |  | *2.5D* | *“Event-driven PrEP is considered as a big stick to do it safe”* |  | |
| **6. Experimenting with the regimen** | Experimenting with the daily PrEP-regimen | 12  (1.4) |  | Experimenting with the daily regimen | 1  (1.1) |  | Experimenting with the event-driven PrEP-regimen | 1  (<1) |  | Experimenting with the daily regimen | 1 (1.2) | |
| *2.6A* | *“First see how body reacts on continuous intake”* |  | *2.6B* | *“[I] want to know what it is like to take it [PrEP] daily. How will it influence my behaviour?”* |  | *2.6C* | *“Try to see if this is the right schedule, first experience [it]”* |  | *2.6D* | *“[I] would like to see if the event-driven schedule is feasible for me”* |  | |
| **7. Other** | Daily PrEP provides solidarity with daily medication users | 7  (<1) |  |  | 0 |  | Event-driven PrEP is cheaper | 1  (<1) |  |  | 0 | |
| *2.7A* | *“Would like to take the pill together with steady partner, he is HIV-positive”* |  |  |  |  | *2.7B* | *“Event-driven PrEP is cheaper”* |  |  |  |  | |

PrEP = pre-exposure prophylaxis; MSM = men who have sex with men; TGSM = transgender persons who have sex with men; cART = combination antiretroviral therapy.

^†^Quotes were originally in Dutch. Dutch quotes have been translated into English.

^‡^Since participants reported at least one reason to choose and switch between PrEP-regimens , the number of motives analysed is larger than the number of participants.

^§^The number of times a specific motive was reported, not equal to the number of participants. Percentages reflect the proportion of all motives (N=857) reported.

| **Table 3. Motives for temporarily or completely stopping PrEP, frequency of motives, and representative quotations MSM & TGSM temporarily or completely stopping PrEP at follow-up, Amsterdam, 2015-2017** | | | |
| --- | --- | --- | --- |
| **Motives for temporarily stopping daily PrEP use (>3 days) (N=161) among 95 participants**^†^ | **n (%)**^‡^ | **Quote nr.** | **Representative quotes**^§^ |
| 1. Adherence and aversion issues | 71 (44.1) | 3.10A | “*Lost them on holiday”* |
| 2. Temporary reduction in risk circumstances | 51 (31.7) | 3.11A | *“I was in the countryside where there are no gays, so also no chances on having sex”* |
|  |  | 3.11B | *“I had an STI so I was not allowed to have sex”* |
|  |  | 3.11C | *“Temporarily had no sex besides steady partner”* |
|  |  | 3.11D | *“[I used] the pills around sexual episodes”* |
| 3. Sickness or poor health conditions | 30 (18.6) | 3.12A | *“[I] needed surgery and was advised to stop briefly”* |
|  |  | 3.12B | *“[I] was not feeling well so [I] stopped with PrEP until I recovered”* |
| 4. Side-effects and physical burden issues | 8 (5.0) | 3.13A | *“[I] had other daily medication, [I] did not want to burden myself too much”* |
| 5. Wanting to control sexual risk behaviour | 1 (<1) | 3.14A: | *“Stop [with daily PrEP] in order to have solely protected sex”* |
| **Motives for stopping PrEP use completely (N=34) among 21 participants**^¶^ | **n (%)**^‡^ | **Quote nr.** | **Representative quotes**^§^ |
| 1. No more need for PrEP | 20 (58.8) | 3.15A | *“[I am] not at risk of HIV anymore”* |
|  |  | 3.15B | “*The idea that you take medication for something you can also use condoms for”* |
| 2. Unacceptable side-effects | 10 (29.4) | 3.16A | *“Participant wasn’t able to enjoy sex due to side-effects”* |
| 3. Other | 2 (5.9) | 3.17A | “*Obliged to stop due to disability insurance for self-employed persons, otherwise not insurable”* |
|  |  | 3.17B | “*He notices that he sought more extremes due to several circumstances, PrEP was one of them”* |
| 4. Aversion against daily medication | 1 (2.9) | 3.18A | “*Aversion against daily medication”* |
| 5. Dissatisfaction with study procedures | 1 (2.9) | 3.19A | “*The too-long questionnaires*” |

PrEP = pre-exposure prophylaxis; MSM = men who have sex with men; TGSM = transgender persons who have sex with men; STI = sexually transmitted infection

^†^Participants reported only one reason to temporarily stop PrEP. The number of motives analysed is therefore equal to the total number of participants that temporarily stopped PrEP.

^‡^The number of times a specific motive was reported, not equal to the number of participants. Percentages reflect the proportion of all motives (N=658) reported.

^c^Quotes were originally in Dutch and have been translated into English.

^¶^Since participants reported at least one reason to stop PrEP, the number of motives analysed is larger than the number of participants
